# Quantifying the Resilience of a Healthcare System: Entropy and Network Science Perspectives

**DOI:** 10.3390/e26010021

**Published:** 2023-12-24

**Authors:** Désirée Klemann, Windi Winasti, Fleur Tournois, Helen Mertens, Frits van Merode

**Affiliations:** 1 Department of Gynecology and Obstetrics, Maastricht University Medical Centre+, Maastricht University, 6229 HX Maastricht, The Netherlands; fleur.tournois@mumc.nl; 2 Care and Public Health Research Institute, Maastricht University, 6200 MD Maastricht, The Netherlands; f.vanmerode@maastrichtuniversity.nl; 3 IQ Healthcare, Radboudumc, 6525 EP Nijmegen, The Netherlands; w.winasti@etz.nl; 4 Elisabeth-TweeSteden Ziekenhuis, 5022 GC Tilburg, The Netherlands; 5 Executive Board, Maastricht University Medical Centre+, 6229 HX Maastricht, The Netherlands; helen.mertens@mumc.nl; 6 Maastricht University Medical Centre+, 6229 HX Maastricht, The Netherlands

**Keywords:** quality of healthcare, entropy, network science, requisite variety, complexity, FRAM, Safety-II, organizational design strategy

## Abstract

In this study, we consider the human body and the healthcare system as two complex networks and use theories regarding entropy, requisite variety, and network centrality metrics with resilience to assess and quantify the strengths and weaknesses of healthcare systems. Entropy is used to quantify the uncertainty and variety regarding a patient’s health state. The extent of the entropy defines the requisite variety a healthcare system should contain to be able to treat a patient safely and correctly. We use network centrality metrics to visualize and quantify the healthcare system as a network and assign the strengths and weaknesses of the network and of individual agents in the network. We apply organization design theories to formulate improvements and explain how a healthcare system should adjust to create a more robust and resilient healthcare system that is able to continuously deal with variations and uncertainties regarding a patient’s health, despite possible stressors and disturbances at the healthcare system. In this article, these concepts and theories are explained and applied to a fictive and a real-life example. We conclude that entropy and network science can be used as tools to quantify the resilience of healthcare systems.

## 1. Introduction

Although the impact of poor outcomes of medical care is immense, attempts to prevent these outcomes often fail. Processes and features in healthcare are complex: there are usually multiple professionals involved, the clinical presentation of diseases may be different and complex, and there is often a need (or feel) to act in a limited amount of time. Clinical decision making ultimately relies on experience and recognition-primed decision-making [1,2].

The quality of healthcare used to be assessed by healthcare outcomes and the occurrence of adverse events and incidents [3]. In addition, more recent methods, such as the Safety-II perspective, aspire to bring focus into situations where safety and quality are actually present. Central to this perspective is the awareness that in complex systems such as healthcare, safety is a consequence of collective efforts to adapt to dynamic conditions and uncertainty [4]. The approach of the human body as a ‘complex (network) system’ is in line with this, which explains how physiological (sub)systems interact to generate a variety of physiological states, to optimize the organisms’ functioning, and to maintain health [5]. A healthcare system should be able to match this complexity.

Healthcare systems should feature a mechanism to (re)act on any change in human health [6]. In 1988, Karl Weick linked healthcare systems to the ‘Law of requisite variety’ [7], as developed by Ashby in 1958. This law describes that for any system to be stable, the number of states that its control mechanism is capable of attaining (its variety) must be at least equal to or greater than the number of states in the controlled system. Weick pioneered analyzing the healthcare system as a network and introduced concepts like ‘enactment’ and ‘requisite variety’ to explain how organizational structures and characteristics influence the quality and safety of healthcare. He argues that the variety of a healthcare system should be at least as extensive and complex as the human body. This approach of Karl Weick is qualitative and descriptive; a tool to measure the quality of strength of (individual) parts of the healthcare system, which could be used to improve the quality of care in a quantified manner, is lacking.

To develop a method to quantify the healthcare system as a network to match the complexity of the human body, we follow Karl Weick’s ideas regarding enactment and (Ashby’s law of) requisite variety [7,8,9] with network science and entropy theory. This enables us to determine the minimum required requisite variety of a healthcare system, and to quantify the strengths and weaknesses of (the individual agents in) a network. Second, we formulate improvements and explain how a healthcare system might increase its resilience by approaching the healthcare system as an information-processing system by applying Galbraith’s [10] organization design theory. We will elucidate our theory, guided by a fictive and a real-life case, previously used by Karl Weick [11], to discuss a practical application of our theory and analyses, showing the implication of this approach for medical practice and clinical research.

## 2. Quantifying Complex Healthcare Systems

In this section, we introduce network and system science concepts relevant for our analysis and suggest a practical tool to apply to healthcare systems.

### 2.1. Health and Healthcare as Complex Systems and Network Science

We consider the human body and the healthcare system as two complex systems that we conceptualize as networks. To understand a complex system, we need to know its components and how they interact with each other [12].

#### 2.1.1. The Human Body as a Complex System and Its Resilience

Representing the human body as a complex (system) network is a topic of current interest and has been utilized in a number of scientific disciplines and research. For example, there is the framework of network physiology, a transdisciplinary research approach that considers the human organism as an evolving complex network—a radically simplified description where the full system (the human body) is described by an interaction network, whose vertices represent distinct physiological *subsystems* (such as organ systems) and whose edges represent time-dependent, observation-derived interactions between them [5]. This can be considered a multigraph, meaning that it is possible to have multiple, parallel edges between subsystems and the subsystems may be connected by more than one edge [13]. Realistic models of biological networks like these require multiple feedback signals, non-linear component dynamics and numerous uncertain parameters [14]. The different organ systems and subsystems may influence and affect the functioning and outcome of other subsystems. This may or may not be visible on a macroscopic level (the human body). The interaction between the subsystems is emergent; outcomes and results on the level of the human body are not apparent or predictable from a detailed knowledge of a system’s microscopic constituents. In other words, it is not just a sum of the individual subsystems, and the functioning of the human body cannot be derived linearly from the sum of the subsystems [15].

The human body tries to maintain a physiological state within certain control limits, called its *homeostasis*. A large disturbance may push a patient’s health state over a critical threshold, pushing it into a new state (both ‘better’ or ‘worse’). In- or external perturbations may temporarily or definitively disrupt this homeostatic state. The *robustness* of the body is the ability to resist deviation from the original state to another health state. The *resilience* of the body is the ability to recover after a deviation occurred [16,17]. The impact of disturbances on a network depends on the stability of the network. When a network is in a stable state, disturbances tend to disrupt the equilibrium only temporarily as the network tends to return quickly to the previous state once the disturbance passes. Illness or malfunctioning of one subsystem will affect the outcome of other subsystems more strongly if those other subsystems have a lower resilience. Thus, if individual subsystems become less resilient, the sensitivity to disturbances increases, which may lead to a cross-correlation between ups and downs in the functioning of other subsystems, making the system less resilient on a macroscopic level, even before the macroscopic system is quantified as ‘unhealthy’ [18].

*Requisite variety* (internal or external) is what is needed to recover to a healthy stage after disturbances from in- or outside the body. If the body fails to recover by itself, healthcare services may provide external requisite variety (see Table 1).

Although the network perspective already provides valuable insights regarding interactions and feedback mechanisms within the human body, this approach also faces several challenges, such as the need for the simplification and parametrization of processes, gathering knowledge regarding the system’s equations of motions, how to merge these equations, and about, for example, relevant structural connections [5]. Thus, a precise network representation of the human body still seems a promise for the future. Nevertheless, the application of network models has already provided valuable insights in, for example, the interaction between organ systems and functions [19] and regarding the robustness and resilience of the human body in healthy and diseased states [16,20].

#### 2.1.2. The Healthcare System as a Complex System

*The healthcare system* is a complex network as well. Nodes/agents represent objects (for example, the people who deliver healthcare, their resources or supplies). Edges are the lines that connect the nodes, representing the association/communication between them [21]. The healthcare system is considered to be *an emergent* multigraph network as well [13].

The cooperation and interaction between patient(s), healthcare workers (doctors, nurses, supporting staff) and supplies lead to results that are not just a sum of the individual contributions of the healthcare workers. The autonomy of the healthcare staff, decentralized decision making and the self-organizing capacity of the healthcare system contribute to emergent feedback loops [22]. The ability of a healthcare system to adjust its functioning prior to, during, or following changes and disturbances in a patient’s health so that it can sustain the required performance (namely, good quality of healthcare) is the *robustness* of the healthcare system. A healthcare system that aims to diagnose and treat low-frequency, complex healthcare conditions must be robust. The healthcare system should provide an iterative diagnostic process by offering enough degrees of freedom for the healthcare workers involved, combined with feedback loops to correct potential wrong (differential) diagnoses), thereby reducing the chance of an error or mistakes. A healthcare system is *resilient* if it is able to recover and maintain a good quality of healthcare after disruptions to the healthcare system, for example, a lack of staff or supply [23]. A more robust and resilient healthcare system is less prone to errors and adverse events.

When a patient approaches a hospital for medical care, the two complex systems (the patient and the healthcare system) have to connect and interact with each other and might (temporarily) merge. The patient’s health state determines the minimum required *requisite* variety: what is needed from the healthcare system in terms of quality, complexity and capacity to regain health.

Communication and collaboration of the different agents in a healthcare system are essential to creating a high-quality healthcare system with the required amount of requisite variety to intervene in the patient’s health condition. Expertise coordination practices, such as reliance on medical protocols, the community of practice structuring and sharing of knowledge, are essential to ensure the timely application of necessary expertise and high-quality healthcare [24].

#### 2.1.3. Centrality Metrics of a Healthcare System

The strength of a healthcare system is determined by the fitness of the individual agents (see Appendix A.1, Appendix A.2 and Appendix A.3) and the cooperation qualities of the agents within the network. The fitness of an individual healthcare worker is determined by their actual competence (such as knowledge and skills), their position in the system and their skills to provide and maintain information processes regarding the patient’s health (change) [12].

The characteristics of an agent (node) *in a network* can be calculated by the *vertex in-degree*, *closeness centrality* and *betweenness centrality* (see Appendix A.1, Appendix A.2 and Appendix A.3). Urata and Hato [25] explain the importance of maintaining information processes between nodes, forming a feedback loop after sending or transmitting a signal. In order to keep a system under control, a closed loop shaped by feedback mechanisms is required. Without feedback mechanisms, an open-loop system occurs, causing possible delays in utilizing information and thereby decreasing the robustness and the requisite variety of the healthcare system [26].

Thus, a node capable of maintaining vertices and feedback loops is a ‘fit’ node. Other important characteristics of a healthcare provider in a network are availability and reliability. Availability and reliability are related to time and precision, two core dimensions for assessing network variability [27].

### 2.2. Quantification of the Robustness and Resilience of the Healthcare System

Each agent in a healthcare system has its own robustness and resilience, which influences the system’s aggregated characteristics. The agent’s position in a network determines how effective the agent is at spreading information and influencing other agents. Previous studies have indicated that a robust and resilient system contains four interrelated abilities: monitoring, anticipating, responding, and learning [28].

In their study to identify key agents in social interactions between healthcare providers, Bertoni et al. [28] use social network analysis. The key players (or agents) are those with an optimal position to quickly diffuse information, attitudes, behaviors, or goods, and quickly receive the same [29]. For each of the four abilities, a score is proposed for each agent, combining five indicators theoretically connected to robustness and resilience: (1) in-degree, (2) closeness, (3) betweenness, (4) availability, and (5) reliability. Given a graph where G = (N, E), N is the set of nodes or vertices and E is the set of edges (i.e., links between vertices). *n* is the number of nodes or vertices, and *m* is the number of edges, and $deg\left(v_{i}\right)$ is the number of edges incident to the vertex. The *in-degree* ( $C_{D}\left(v\right))$ , *closeness* $\left(C_{\left(v\right)}\right)$ and *betweenness* ( $C_{B}\left(v\right))\;$ are explained in the text box and can be calculated with the formulas discussed in Freeman [30] and Bertoni [28] et al.’s works, as follows:

*In-degree* of vertex *v* ( $C_{D}\left(v\right)$ ):

(1) $$C_{D}\left(v\right)=\deg v$$

*Closeness* of vertex *v* ( $C_{\left(v\right)})$ :

(2) $$C_{\left(v\right)}=\frac{N-1}{\sum_{v}d\left(s,\;t\right)}$$

where d(s, t) is the distance between node *s* and *t*.

*Betweenness* of vertex *v* ( $C_{B}\left(v\right))$ :

(3) $$C_{B}\left(v\right)=\sum_{s\neq v\neq t\in N}\frac{\sigma_{st}\left(v\right)}{\sigma_{st}}$$

where $\sigma_{st}$ is the number of shortest paths from node *s* to node *t*, and $\sigma_{st}\left(v\right)$ is the number of shortest paths from node *s* to *t* that pass though vertex *v*.

The *availability* impacts the variability in the time dimension since agents who are not available may delay information transfer. The *reliability* impacts the variability in the precision dimension since incomplete or imprecise information compromises actions and decisions based on social interactions. The availability and reliability of an agent are non-network attributes, and may be assessed using Likert-style questions and scored using a five-point scale (1—never to 5—always) [28]. To eliminate differences in the outcome (scales) of these metrics, Bertoni et al. rescale all outcomes into a 1-to-5 scale.

Following Bertoni et al. [28], the robustness and *resilience* score ( $RS_{v}$ ) of agent (vertex) *v* in network *j* can be calculated as follows:

(4) $$RS_{v}=\left[C_{D}\left(v\right)*C_{\left(v\right)}*C_{B}\left(v\right)\right]*A_{v}*R_{v}$$

where $C_{D}\left(v\right)$ , $C_{\left(v\right)}$ , and $C_{B}\left(v\right)$ are the in-degree, closeness and betweenness of the agent, rescaled onto a 1-to-5 scale. $A_{v}$ is the average availability score, and $R_{v}$ the reliability score. The score of each agent can be measured for each ability (monitoring, anticipating, responding, and learning).

A healthcare system itself gains robustness and resilience by applying closed loops between the agents involved (see Section 2.1.2).

### 2.3. Entropy

To quantitatively characterize networks and objectify the minimum requirements of a healthcare system to deliver the needed requisite variety to heal a patient, *entropy* must be taken into account. Entropy is a measure for ‘uncertainty’ and was designed for the perspective of the probabilities of different configurations (microstates) of particles in an ensemble (macrostate) (see Appendix A.4, Appendix A.5, Appendix A.6, Appendix A.7 and Appendix A.8).

Translated to healthcare, the patient is the *macrostate* that ‘men’ (the doctors in the healthcare system) can see. The underlying (combination of) health states or disruptions of health states are the *microstates*. A healthcare worker interprets signals from the patient to diagnose a macrostate. There will always be a certain degree of uncertainty between the perceived (diagnosed) health state and the actual underlying microstate(s). This uncertainty of the healthcare professional about the true health state is *the entropy of the human body*. Entropy is in the eye of the beholder, and thus the decision maker’s perspective at the moment of decision making. As a network (see Section 2.1.1), the human body’s entropy can in principle be determined as follows [31]:

(5) $$H_{graph}=-\sum_{i=1}^{n}\frac{deg\left(v_{i}\right)}{m}*\log_{2}\frac{deg\left(v_{i}\right)}{m}$$

The value of the entropy lies in the interval $\left[0,\log_{2}n\right]$ .

A competent healthcare provider (a fit agent of the network) has an interpretation framework available, based on education, training, experience and protocols, to ‘decode’ the signals (symptoms) sent by the patient and formulate differential diagnoses. The assessment of the health state of a patient contains a degree of subjectivity influenced by the reference framework of the assessor. A list of differential diagnoses will lead to actions, such as using diagnostic tests, referring to other doctors or treating a (probable) diagnosis. These actions are placed into a certain sequence, defining the patient’s path. This path also defines the competence and capacity requirements for the healthcare system. A patient’s path and the capacity path of the healthcare system form the interdependence between the demand and supply of healthcare. As more information becomes available regarding the patient’s health, the number of feasible options reduces and thus the entropy and the required requisite capacity of the healthcare system also decrease.

It is important to note that the *entropy* regarding the *microstate* of the patient should be distinguished from the *variety* of the health state of the same patient. Suppose a patient has multiple symptoms related to different organ systems. In that case, there is a bigger variety of health states and differential diagnoses, requiring a higher variety needed of healthcare providers’ competences, diagnostic tests and possible treatments. If the variety increases, so does the entropy. The variety and the uncertainty (entropy) regarding the patient’s health determine the requisite variety of the healthcare system to intervene in the patient’s condition to heal it. Both the uncertainty and the variety are incorporated in the algorithm to calculate the entropy of a network following Monostori since the numeration of the fraction ( $deg\left(v_{i}\right)$ ) (Formula (5)) corresponds to the possible manifested health states.

Other forms of entropy, such as ‘project entropy’ and ‘positional entropy’ [32], could be calculated and incorporated as well but are not included in our hypothesis since they might have a smaller impact on the requisite variety of a healthcare system in relation to the resilience of the human body.

### 2.4. Information Processing

Following the above, if a patient’s condition is curable, the outcome of this particular patient depends entirely on the availability of the minimum required requisite variety of the healthcare network to compensate the lack of inner resilience of the patient.

If the potential requisite variety of the healthcare network is not realized, this is due to a lack of competence of one or more agents (healthcare providers) in the network, or a lack of information processing within the healthcare system (interaction, collaboration and communication). To a certain degree, information processing may compensate for the lack of competence of one or more agents, and vice versa. Information processing contributes to both the robustness and the resilience of the healthcare system. Specific agents (nodes in the network) may fulfill their role as information processors, depending on their fitness (both their competence and strength and position in the network).

The increase in subspecialists, due to increased knowledge, techniques and possibilities in current healthcare, makes it impossible for one doctor to have the knowledge and skills to diagnose and treat every possible health condition of a patient. An unavoidable disadvantage of this (sub)specialization is the risk that specialists will focus on the diagnosis and treatment of a medical condition within their own specialty without seeing ‘the bigger picture’, possibly leading to risks for the patient’s health. Hospitals and subspecialists must balance the differentiation and integration of knowledge in patients with complex pathology [33]. This requires collaboration between specialists and care coordination for patients with complex, multi-organ pathology. This is a problem of ‘organization design’ [10].

### 2.5. Organizational Design Theories

Galbraith [10] defines coordination as information processing to improve organization design, determining that either the need for information (processing) should be reduced or the information-processing capacity should be increased. A reduction in the need for information processing can be realized by creating slack resources or self-contained tasks within the organization. In contrast, an increase in information-processing capacity can be realized by investing in vertical information systems or creating lateral relations.

Slack resources in healthcare are often viewed in a negative way (for example, as ‘waste’). However, slack resources and redundancy are essential in facilitating flexible, continuous and safe healthcare [34]. Slack resources, such as redundant or multi-employant staff, are a form of ‘free energy’. The ‘free energy’ principle is a theoretical framework applied in biophysics and cognitive science. It is as mathematical principle that suggests that ‘free energy’ accounts for action, perception and learning. Free energy is necessary for process optimalization [35]. An increase in free energy contributes to a more robust and resilient healthcare system.

The creation of self-contained tasks in healthcare could be realized by referring to a doctor capable of treating the ‘whole patient’, a system doctor with integrated knowledge, thereby reducing the need for information processes. In reality, such doctors do not exist. Investment in vertical information systems means that previous plans, events and solutions should be revised more frequently. A medical example of a vertical information system is the formation of a ‘care path’, in which one doctor, for example, the general practitioner, is named as the ‘head practitioner’ and is in charge of the coordination of care. The general practitioner is, in that case, not only the agent that refers the patients to a (sub)specialist, but he also coordinates the health care between the other agents. Informational feedback loops are indispensable within vertical information systems since these deliver the information to the general practitioner. Last, the creation of lateral relationships includes moving the level of decision making down in the organization to where the information exists. Examples of the lateralization of relationships are direct contacts between agents that share a problem, liaison roles (specialized to facilitate communication), task forces and teams. A medical example of a lateral information system is the formation of a subnetwork of healthcare providers, for example, a team of subspecialists from different disciplines that cooperate. Forming a (periodic) multidisciplinary consultation can be considered horizontal integration of information. The formation of a temporarily subnetwork can be considered pooling: the participating agents as the nodes of the subnetwork are aggregated to one new agent. By pooling the agents, the need for information processing and entropy decreases [32].

Knowledge regarding the information processes within a network and the fitness of the agents involved can be quantified by measuring the resilience of the individual healthcare workers. This helps to point out which players are crucial and which changes in the interaction should be made to improve the resilience and thereby the safety and quality of healthcare.

### 2.6. Summary

A diseased patient might seek help from a healthcare system to heal. In network terms, an in- or external perturbation may have caused a disruption of a health state and the body lacks inner resilience. The complexity and the extent of interventions needed from the healthcare system are the requisite variety. There is always a certain degree of uncertainty regarding the patient’s healthcare condition (the true microstates causing the perceived diagnosis). This uncertainty, combined with the variation in symptoms of the patient, is called the entropy of the human body. This entropy inherently influences the required complexity and extent of the healthcare system to treat the patient. The quality of the healthcare system depends on the accuracy (knowledge and skills) of the individual healthcare providers and the information processing between them. To continuously provide a ‘high quality of care’, the healthcare system should be *robust* to changes in the patient’s health state, and resilient to in- and external perturbations of the healthcare system itself and previous mistakes of the healthcare system. Applying centrality metrics to the healthcare system as a whole, and robust- and resilience scores to the individual healthcare providers results in insight regarding the strengths and weaknesses of the system and the ability to correct previous mistakes. This information, combined with organization design theories translated to network concepts, can be used to improve the resilience and thus the safety and quality of a healthcare system.

Next, we will provide a step-by-step application of this theory to a fictive and a real-life example.

## 3. Step-by-Step Application of Theories

We will demonstrate the theory mentioned above by analyzing the provided healthcare to a (fictive) patient. All figures, calculations and analyses have been made with Mathematica 13.3 [36].

### 3.1. Case Study

**Figure 1:**
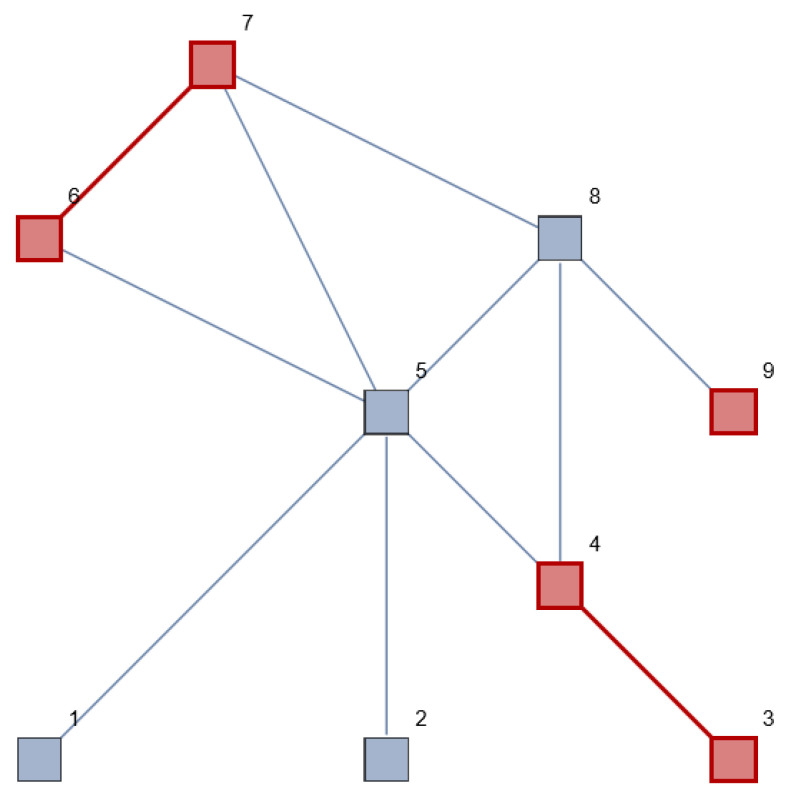
System/network view of the human body, with multiple vertices (elements of the human body), connected to each other by edges. The symptoms (red, no. 3 + 4, 6 + 7 and 9) are interpreted by the GP and classified into three diseased organ systems that seem to have no direct connection to each other.

A patient visits their general practitioner (GP) and mentions five symptoms, for example, muscle strain, dyspnea, coughing, abdominal pain and diarrhea. The symptoms are interpreted by the GP, who classifies these symptoms as diseases from three different organ tracts (for example, the respiratory system, the digestive system and the muscular system). The GP assumes a certain macrostate of the patient (diseased) and makes a list of differential diagnoses (microstates) that might cause these symptoms. Figure 1 represents the patient (although the subject, content and application are different, we followed Tang et al.’s work [37] as an example for the network structure). The red nodes and edges represent the patient’s symptoms, classified and clustered by the GP into three separate organ tracts. He does not expect an overarching diagnosis that causes all the symptoms in the different organ tracts. The elements are connected to each other and are all part of the human body.

The list of differential diagnoses the GP assumes and the diagnostic/therapeutic steps that are taken determine the patient path. Let us assume the GP in this case refers the patient to three different medical specialists for each organ tract involved. Suppose the symptoms are indeed treated independently from each other by the different (sub) specialists, but, in reality, there is a non-observable connection between these symptoms (another, not yet recognized, overarching microstate), and the disease of the patient is undertreated because each specialist only observed, diagnosed and treated a part of it. The variety of symptoms and the uncertainty regarding the true microstate causing these symptoms is the *entropy*, as mentioned before. The symptoms mentioned by the patient define the complexity of the disease and treatment for the physicians involved. The *potential* complexity, however, is the complexity of an unrecognized, or un(der)treated disease.

**Figure 2:**
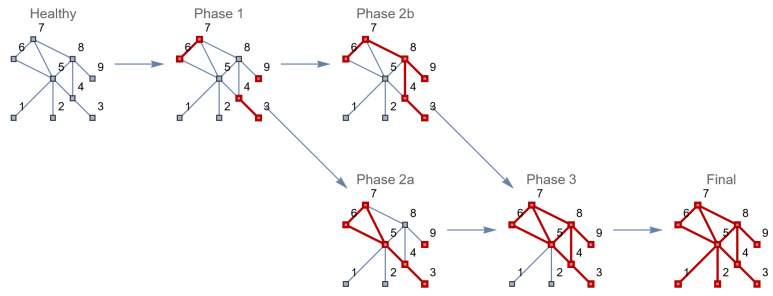
Transition of a healthy human to a patient with separate diseased elements (phase 1–2b) and a systemic disease (phase 3).

What might happen if these disease subnetworks have a chance to influence other (neighboring) nodes in the network, and thus also the edges between these nodes? The disease might then evolve and become a systemic disease, as shown in Figure 2.

A wrong diagnosis and/or a wrong referral and treatment may lead to the *enactment* of another disease state of the patient due to an insufficient requisite variety of the healthcare system. This insufficiency may be caused by the incompetence of the individual healthcare providers (in this case, of the GP and/or the medical specialists), patient-related circumstances or (a lack of) interaction with other healthcare providers leading to uncertainties, wrong referrals, diagnosis and treatments. If a closed (information) loop is not provided, no one will see the lack of the required response to the started treatment and the patient’s healthcare will only become worse.

The *variety* and *uncertainty* of the human body determine the entropy regarding the true microstate(s) of the patients, which defines the *requisite variety* of the healthcare system treating the patient.

### 3.2. Entropy of a Human Body

The *entropy* of the human body (composed of the variety of symptoms and the uncertainty regarding the underlying microstate) can be calculated for each health state (see Table 2). To calculate the entropy, we used Formula (5).

When the patient’s health condition deteriorates (from a multiple organ disease to a system disease), the entropy decreases since the possible microstates causing all the patient’s symptoms decrease (increasingly fewer diagnoses will fit all the symptoms). In the end, there is no more entropy (the patient is dead and the health state is known). Thus, a lower entropy in our approach does not always correspond with a better condition of the patient, which means that there is less uncertainty regarding the underlying microstate causing the assumed (diagnosed) macrostate of the patient.

### 3.3. Robustness and Resilience of a Healthcare System

How would this scenario develop in medical practice? The general practitioner will most likely refer the patient to one or more out of three organ specialists (for example, a pulmonologist, general surgeon and a physiotherapist) who might (all) start diagnosing and treating one of the diseased elements), or to a system doctor (a physician who specializes in the total network of a disease). The ability of the healthcare system to correctly diagnose the patient, and to correct/prevent mistakes in the diagnostic process before an error occurs, is the robustness of the system. For simplicity reasons, we replace each (sub) network in the first disease state with one node, representing a possible physician specialized in that specific disease network. If one of the physicians makes a mistake in referring or diagnosing the patient, the disease(s) might evolve to another (systemic) state. The possibility from the healthcare system to recognize and correct a previous mistake is the resilience. Suppose that each doctor (the general practitioner, the organ specialists, and the systemic doctor) can act either rightly or wrongly, as follows:

Doctor 1 (GP)
  - Wrong: refers to one of the three organ specialists.
  - Right: refers to the system doctor.

Doctor 2 (organ specialists; in this case, doctors 2a, 2b, 2c)
  - Wrong: treats one disease element/organ.
  - Right: refers to the system doctor.

Doctor 3 (system doctor)
  - Wrong: does not recognize the disease.
  - Right: treats the disease successfully.

**Figure 3:**
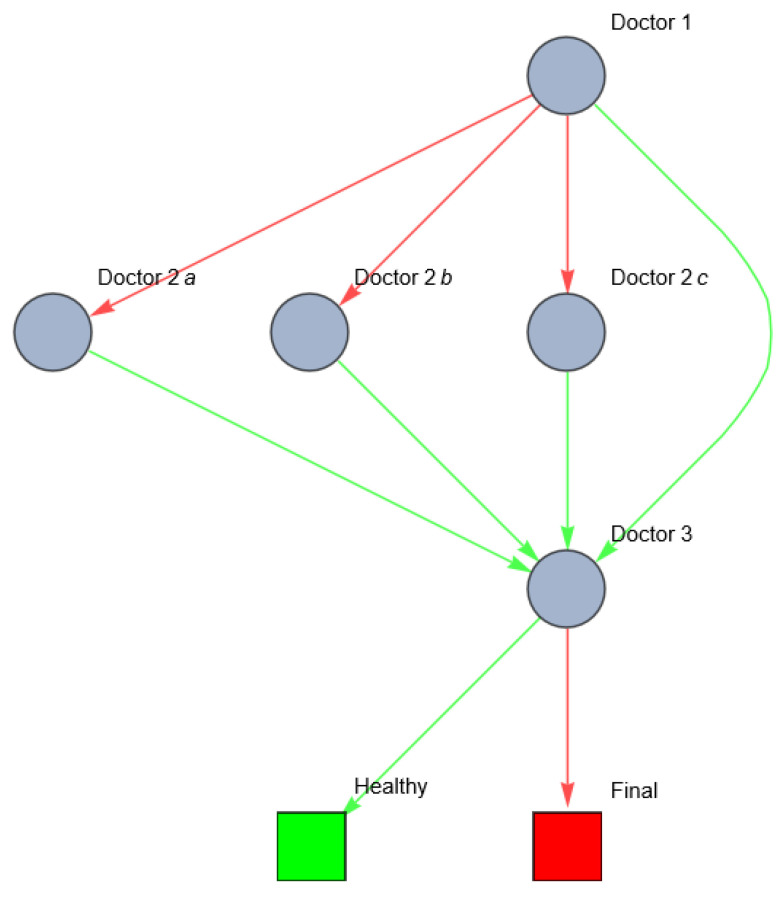
Network of the possible patient pathways in the example above (red: wrong; green: right).

This can be visualized as the following network (Figure 3).

**Figure 4:**
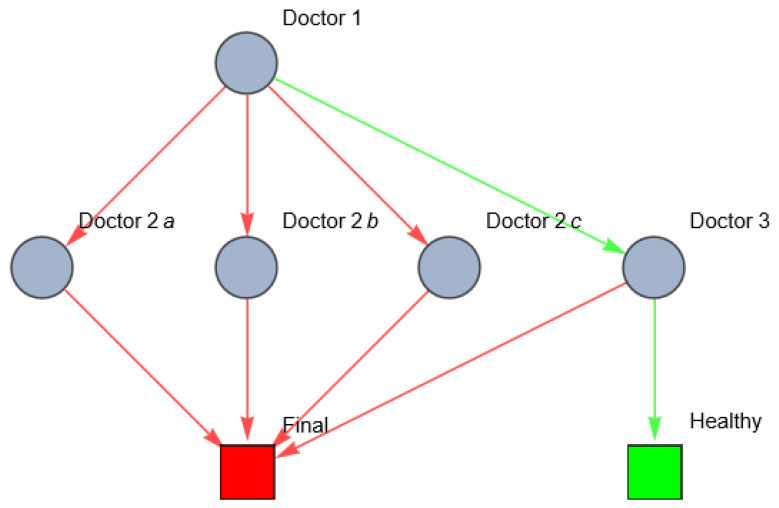
Network of possible patient pathways in the example above with time constraints (red: wrong; green: right).

In real life, patient pathways are even more complex because of time constraints. Suppose that the patient can only survive one referral; the referral should be right the first time. In this case, the network looks as follows (Figure 4).

Diagnosing the right disease state requires process optimization to optimize the limited time available to treat the patient. Not only should the path to the goal state be limited to a certain number of edges, but the nodes (healthcare workers) should also have a certain fitness to diagnose, refer and treat right. If all agents in the network were fully incompetent (zero fitness), there would be maximum entropy of the patient’s possible treatment paths.

Treating the patient’s sickness deals with two characteristics: the *variety,* and the *uncertainty* (thus, the *entropy*). The variety in the previous example can be expressed as the fact that there are five symptoms mentioned by the patient, and as interpreted by the GP, multiple (3) organ systems involved. Each of them can be diseased separately and recognized as such, between one and three organs could be diseased, or there could be one system disease (meaning that there is some emergent disease), that deteriorates all three organs. If we denote *d_i_* as the disease of organ *i*, and *d_s_* as a system disease, we have the following variety of diseases that the doctor is confronted with:

1. D_1_.
2. D_2_.
3. D_3_.
4. D_1_ and D_2_.
5. D_1_ and D_3_.
6. D_2_ and D_3_.
7. D_1_ and D_2_ and D_3_.
8. D_s_.

**Figure 5:**
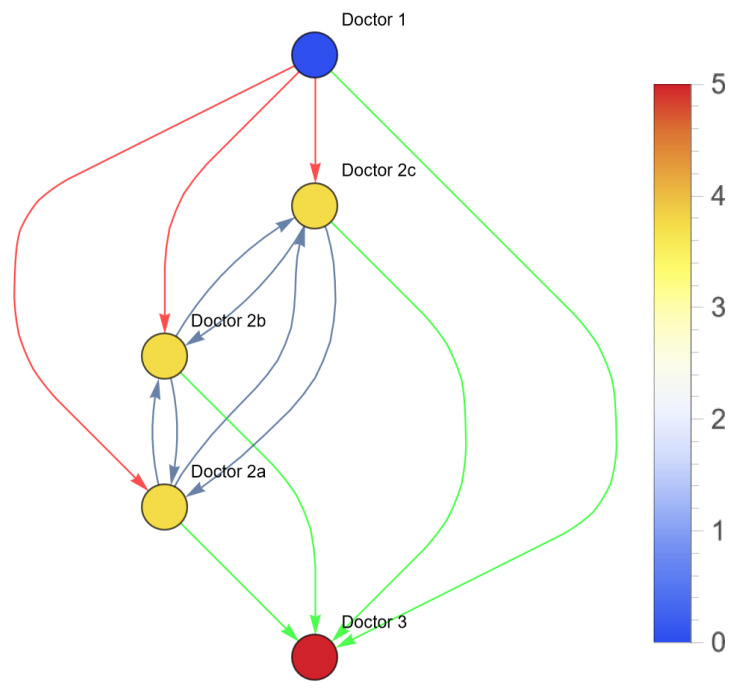
Vertex in-degree of each node in the healthcare system of the aforementioned example. The color of the node (agent) corresponds with the vertex in-degree of this node.

The total variety of healthcare states in this example is eight. The uncertainty of the patient’s sickness concerns the probability of the actual occurrence of each variety. A differential diagnosis hypothesizes a certain occurrence of microstates causing the perceived (and interpreted) macrostate. Whether the right differential diagnosis is selected depends on the signals the doctor receives from the patient, colleagues, diagnostic tests and the competence of the doctor. The process of setting and rejecting differential diagnosis reduces the entropy of the patient’s health. If successful, the variety of possible disorders reduces in each step of the patient’s path, and uncertainty regarding the actual diagnosis decreases. Wrong referrals may lead to further breakdown of the diseased network. This is Karl Weick’s ‘enactment’ (see Appendix A.8). The healthcare system should continuously monitor and adapt to changes in the healthcare system. In order to maintain a high quality of care, despite changes in the patient’s health state and interruptions within the healthcare system, the healthcare system must be robust and resilient. This is determined by the robustness and resilience of the individual agents, and the cooperation and interaction between the agents. This can be quantified using centrality metrics (Formulas (1)–(3)). One of the centrality metrics is the vertex in-degree of the healthcare workers. Figure 5 shows the vertex in-degree of each doctor in the previous example. The vertex in-degree is a measure for the (potential) information input to this agent. Doctor 3 has a higher number of possible information input compared to doctor 1 (see Figure 5).

Figure 5 is illustrative for several reasons. First, we can see the possible information process between nodes (healthcare providers) in a healthcare system. In this example, doctor 1 (GP) seems to have a very low in-degree centrality. This can be explained by the fact that this doctor only refers the patient to other doctors without receiving feedback/information from these agents. If there were a closed-loop system and the GP received feedback from the other doctors (regarding their findings, diagnostic tests and their results and possible treatments), the in-degree centrality of this doctor would increase, and the possible variety of the other paths could be decreased. Second, the in-degree centrality reflects the possible pathways, but the patient’s actual path depends on decisions of each node. Third, doctor 3 seems to have the highest in-degree centrality since the patient might reach him through different pathways. This makes doctor 3 a doctor with a high in-degree centrality, but this does not necessarily mean that this is the fittest doctor, because we do not know if doctor 3 is competent on a medical level, and/or if he is able to remain involved in the treatment of the patient in a sustainable way.

The other metrics of this network can be calculated as well (see Table 3).

### 3.4. Robustness, Resilience and Information-Processing Theory

**Figure 6:**
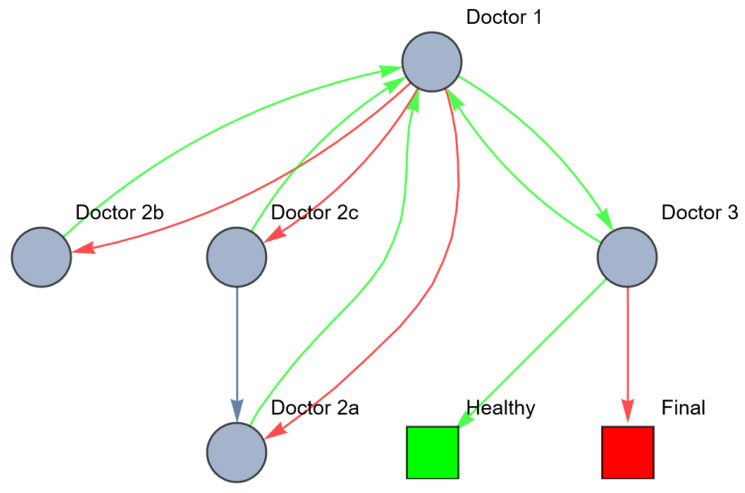
Network of information processing in the example above after investment in vertical information systems (red: wrong; green: right).

In healthcare, robustness and resilience rely on information processes and (Galbraith’s) theories of organization design can be applied to increase these. Investing in vertical information systems is one way to redesign the healthcare system, for example, by appointing the GP as ‘head practitioner’ and coordinator of care. This implies that the GP still has a choice to refer the patient to one or more subspecialist(s) or to the system doctor in the first place. However, after referring, an information feedback loop from every doctor the GP referred the patient to is provided. If the subspecialist(s) performed diagnostic tests or started a treatment, the outcome will be communicated to the GP. The GP will evaluate whether or not the expected outcome or response occurred. If not, the GP will refer the patient to another subspecialist or to the system doctor. Ideally, the subspecialists will not refer the patient directly to each other but only via the GP. This situation can be represented as follows (Figure 6).

We can measure the resilience and entropy of this system as well (see Table 4).

The results in Table 4 show that vertical information systems increase the robustness and resilience of the healthcare system. For example, doctor 1 has now closeness centrality of 1.0 (was 0), meaning that doctor 1 is now also processing information to reduce the variety of the patient’s path (hence reducing the entropy) and thereby increasing the resilience of the healthcare system, enabling it to maintain a high requisite variety, meaning that it has more potential to correctly treat the patient despite disturbances or previous mistakes of the healthcare system itself.

**Figure 7:**
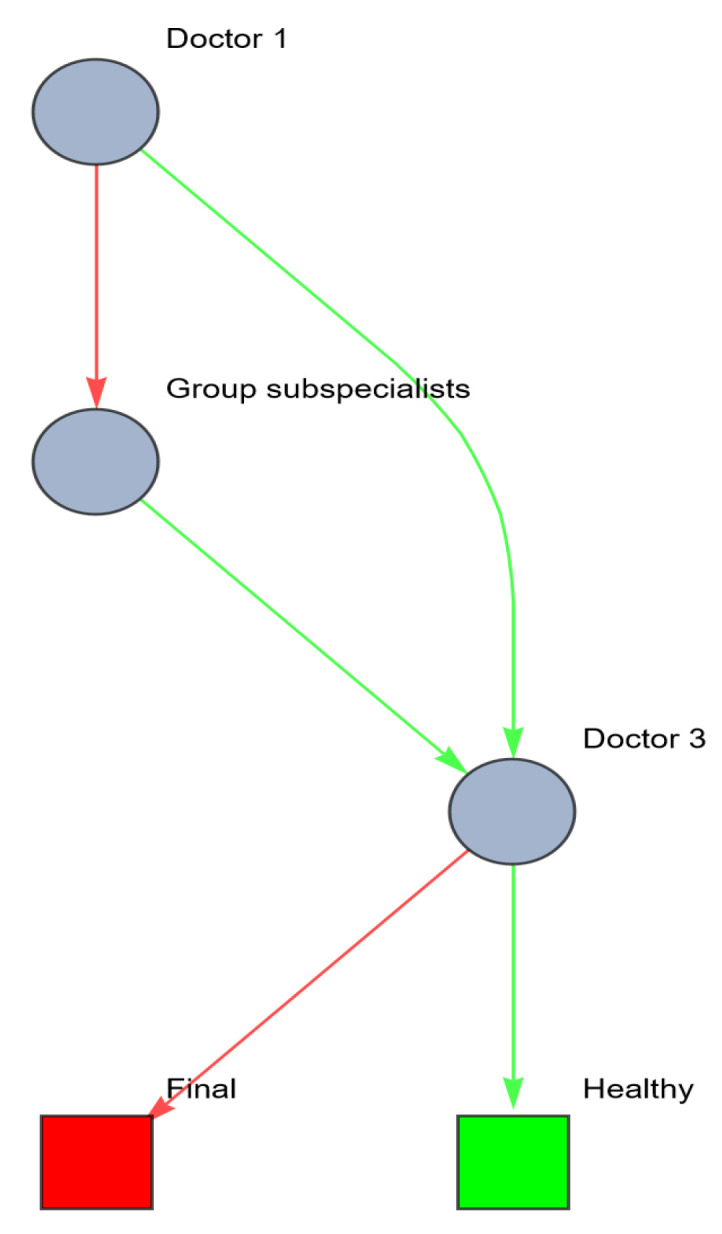
Network of information processing in the example above after investment in lateral information systems (red: wrong; green: right).

Another option to redesign the healthcare system to increase robustness and resilience is by investing in lateral relations between the healthcare providers. In the previous example, after referring the patient to one or more subspecialists, the GP or one of the subspecialists should note that the patient is suffering a systemic disease that affects multiple organ systems. A multidisciplinary consultation between all healthcare providers involved should be generated. In network terms, the cooperating doctors can be pooled (aggregated) to one new agent (the multidisciplinary team), thus reducing the need for information processing and the risk for enactment of the health state. This can be visualized as follows (Figure 7).

Resilience metrics of this network are shown in Table 5. Care must be taken to ensure that the treatment leads to the desired and expected response by periodically evaluating the patient’s health state. If the desired response is not reached, a re-evaluation of the patient’s true health state should follow.

**Figure 8:**
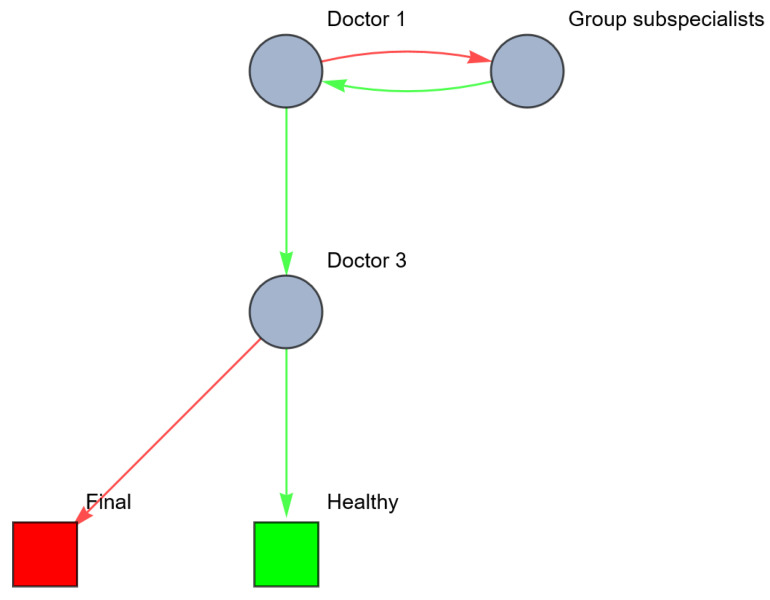
Network of information processing in the example above, after investment in lateral and vertical information systems (red: wrong; green: right).

A variation in this scenario could be a combination of lateral and vertical relations, for example, by running all information exclusively through the GP instead of via the pooled subspecialist. This can be visualized as follows (Figure 8).

The metrics of this network are shown in Table 6.

As mentioned above (Section 2.1.3), besides the centrality metrics, the availability and reliability of each agent should also be taken into account to measure the fitness of an agent [28]. The availability and reliability of an agent are non-network attributes and may in practice be assessed using Likert-style questions. Ideally, each doctor would be available 24/7 and 100% reliable in transmitting and coordinating information. In reality, healthcare providers are not available 24/7 and even if available, they have to take care of multiple patients and can be occupied with other tasks, thus decreasing the availability of each agent. Both the availability and the reliability of the agents are determined by the agents’ personal capabilities (knowledge, skills) and the circumstances of the network.

In the experimental design table below (Table 7), we calculate the impact of the availability (*A_i_*) and reliability (*R_i_*) (scored with values of 1 (low), 3 (average) or 5 (high)) of each agent from the last example (the combination of lateral and vertical relations) on their resilience (as shown in Table 6).

In conclusion, the variety and uncertainty regarding the patient’s health, quantified by the entropy of the network of the human body, define the requisite variety a healthcare system should contain. The resilience of the healthcare system is a measure of the ability to maintain a good quality of healthcare despite disturbances of the healthcare system or previous mistakes in diagnosis or treatment. Theories of organization design can be applied to increase this resilience. Translating these theories to network concepts, we are able to quantify healthcare systems and calculate the expected effect of changes in organization design on the resilience.

Next, we will apply our theories and calculations to a real medical case. The same case was used by Karl Weick to discuss the concept ‘enactment’. We will use this case to demonstrate how our method can provide a starting point for improving the resilience and quality of healthcare.

## 4. Applied to a Complex Medical Case

As mentioned in the introduction, Karl Weick [38] already described the matter of medical errors as a result of system errors instead of (individual) human errors. He used terms like ‘requisite variety’ and ‘enactment’ to motivate researchers to pay attention to natural systems and behavioral structures of interpersonal influence, improvisation and social support. He quotes a medical case from Abernathy and Hamm [39] to illustrate how too many varieties and uncertainties in the patient and too little variety in the healthcare system may lead to an uncontrollable situation. The case reads as follows:

*A man in his late 50s suffered severe chest pain at home; he suspected a heart attack but did not go to the hospital. Thirty-six hours later, short of breath from congestive heart failure and pulmonary edema, he felt bad enough to call an ambulance. He arrived at the emergency department in respiratory failure and was intubated and immediately admitted to intensive care.*

*The cardiologist initiated a heparin drip and a lidocaine drip, routine measure for myocardial infarction. The patient had arrhythmia, and so the cardiologist added a Pronestyl drip. Because of the patient’s pulmonary edema, the respiratory internist was concerned about bronchospasm and added a theophylloine drip.*

*The second day in the intensive care unit, the stress of the crisis and the heparin had affected the patient’s digestive tract. The gastroenterologist scoped him, saw stress gastritis and ordered a Pepcid drip.*

*The patient got worse and worse and lost consciousness. His primary physician called a nephrology consult, who looked at the system, and surmised that the patient had a dilution hyponatremia. Each of the 5 drips was in the stand D5DW solution, and together they were diluting the blood sodium beyond the patient’s capacity to compensate, because of pain, congestive heart failure, and renal failure. Laboratory tests showed that the serum sodium was down to 90 from the normal value of 130. The patient died before the doctors could totally fix the hemodynamic balance.*

**Figure 9:**
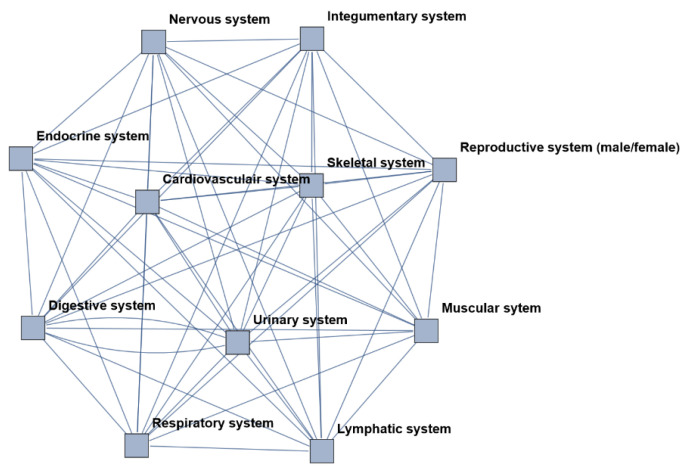
The human body visualized as a network. Each node represents an organ system; the edges represent the connections and correspondence between these nodes. This is a simplified, schematic visualization of reality.

The human body as a network is presented in Figure 9 (see Section 2.1.1). Each node represents an organ system. In a healthy body, all organ systems correlate with each other and maintain a steady state (homeostasis).

**Figure 10:**
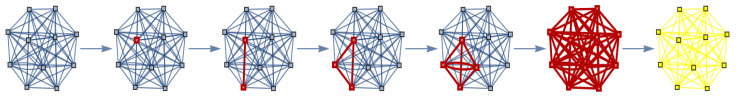
The human body visualized as a network. The blue nodes and edges represent healthy organ systems. The red nodes and edges represent diseased organ systems. Schematic development from a healthy body (left) to a single organ disease, a multiple organ disease and eventually a systemic disease and death (yellow).

In this case, the first symptoms the patient experiences are all related to the cardiovascular system. The cardiologist interprets, diagnoses and treats the cardiac symptoms. Despite (or, in hindsight, as a result of) this treatment, the patient develops new symptoms, first regarding the respiratory tract, then the digestive tract and eventually the urinary tract, before a system disease is recognized. One by one, the organ systems are affected by the treatment for the mistaken diagnosis, leading to an enactment from a one-organ disease to a multiple-organ disease and eventually a system disease (Figure 10).

To analyze and quantify this case as a network, we have to convert the (qualitative) description from above into a table. Therefore, we first scored the following factors (see appendices):

- Agents (A) involved (see Appendix B, Table A1).
- Resources (R) used (see Appendix B, Table A2).
- Organ systems (O) diseases (see Table 8).
- System states (S) of the patient (see Table 9).
- Connections (C) between agents, resources and health states at each moment (see Appendix B, Table A3).
- Differential diagnoses (D) that have been considered by the agents (see Appendix B, Table A4).

Second, we carried out an overview of the situations occurring described by these factors during the development of the case, as shown in Table 10.

**Figure 11:**
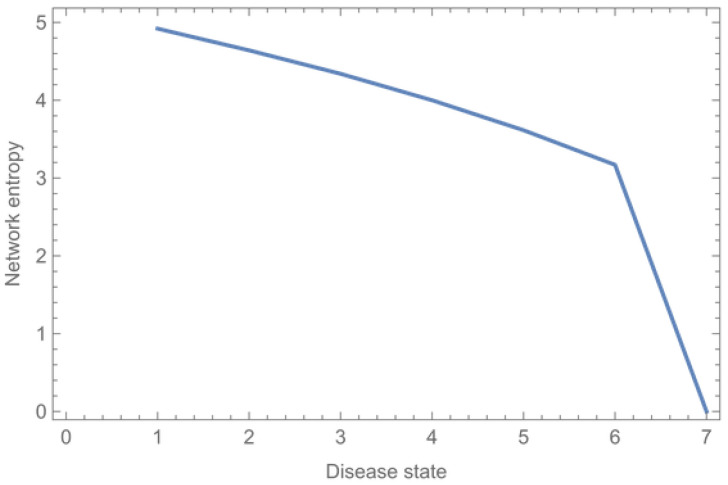
Entropy of the human body in each system state.

As in the previous, fictive example, we are able to quantify the entropy of the body in each system state. This is shown in Table 11 and Figure 11.

Over time, the health state of the patient worsens as more organ systems become affected and the attempts to cure the patient fail. Contrary to what one might suspect, the entropy and uncertainty decrease as the patient’s condition worsens, because organ systems (nodes) and vertices stop working (see Figure 11). When the patient dies, there is no more resilience and there is zero entropy of the body.

**Figure 12:**
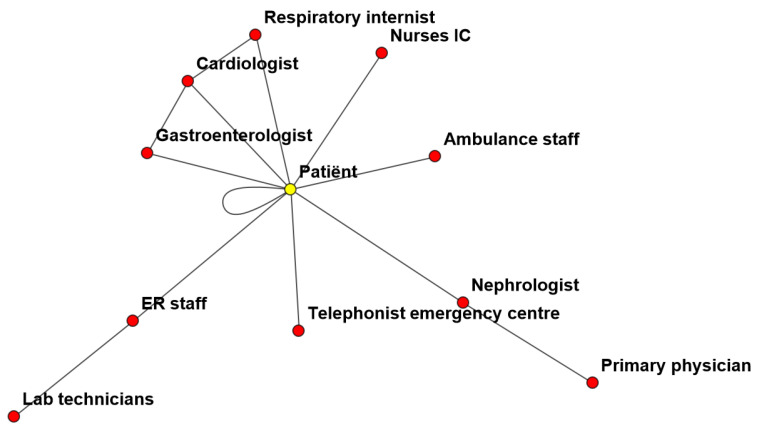
Network of agents involved in this case (nodes) and their information processing (vertices).

The next step in our analysis is to visualize the healthcare system that responds to the patient’s healthcare state(s) and the information processing during the treatment (see Figure 12).

**Figure 13:**
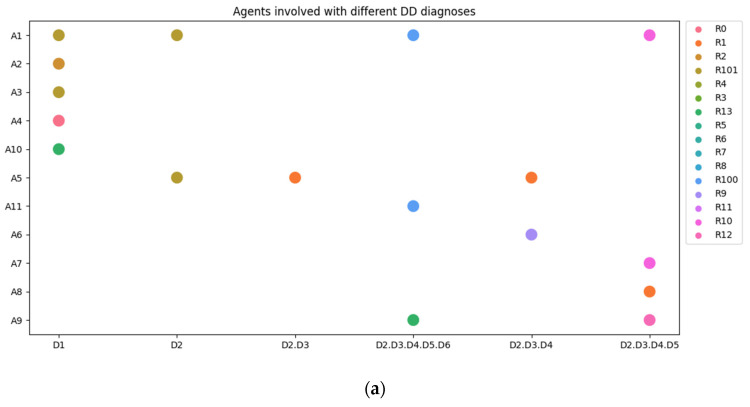

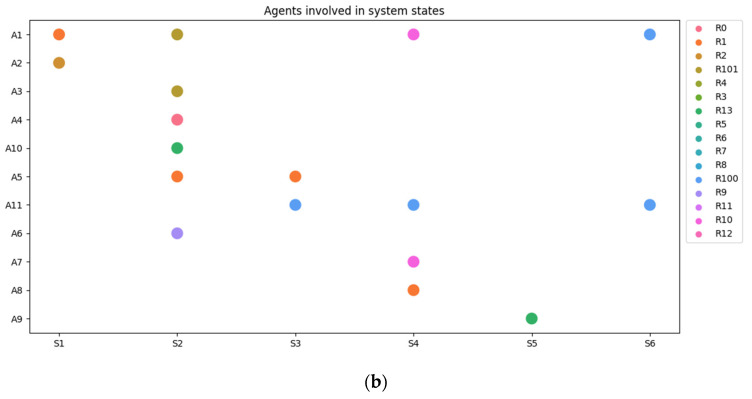
(**a**) Agents and resources involved per differential diagnosis. (**b**) Agents and resources involved per health state.

Remarkable in this network is the large number of agents (doctors and other health providers) and the minimum number of vertices (communication). There is only one central agent with high in-degree centrality, which is the patient itself. It seems that all connections and all communication are carried out through the patient. In the following figures, we show the involvement of all agents per differential diagnosis (Figure 13a) and per system state (Figure 13b).

For analyzing the healthcare organization in this case, we concentrate on the following agents:

- ER staff.
- Cardiologist.
- Respiratory internist.
- Gastroenterologist.
- Primary physician.
- Nephrologist.

**Figure 14:**
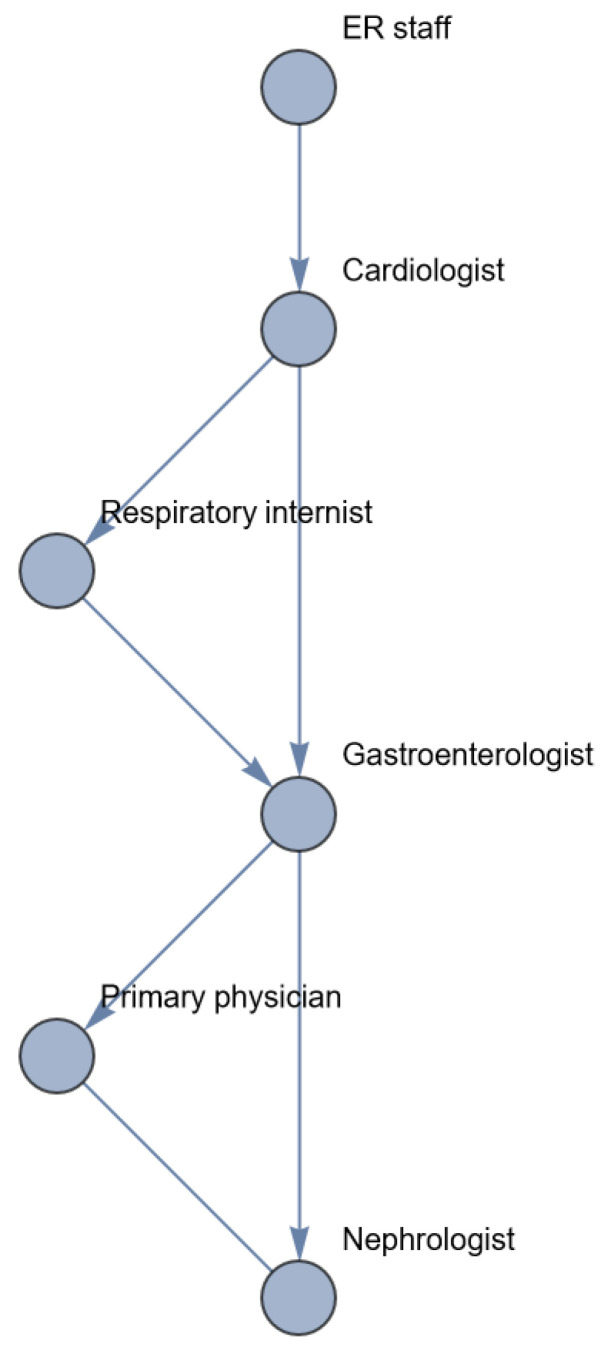
Healthcare network as reconstructed from Karl Weick’s case.

We exclude the telephonist, emergency center, ambulance staff, lab technicians and IC nurses. They are important but are not involved in medical decision making as far as we can judge from the available text. Figure 14 represents the network as reconstructed from the text. Table 12 shows the centrality metrics of this network and its actors.

It is noteworthy that when the patient arrives and the entropy is highest (Table 11 and Figure 11), the lowest number of healthcare providers are involved. As the patient’s condition deteriorates and entropy decreases, the number of healthcare providers increases. In hindsight, no one could see ‘the bigger picture’ and the underlying common cause of all the symptoms until the nephrologist was consulted. At that time, a domino effect had already occurred and the patient’s condition had aggravated too far to be cured. As Karl Weick noted, the physicians created a more complicated patient than each of their specialties could handle. They did so in the mistaken belief that they were merely reacting to the patient’s condition, not enacting it [11].

Comparing the entropy of the patient’s condition and the healthcare system that needs to intervene with this, it appears that when entropy is highest, the number of healthcare providers and the information processing between them is lowest. This discrepancy is the lacking requisite variety of the healthcare system, making it possible for this enactment of health states to occur. Second, when the health state of the patient changes, there is no new, extra processing of information and communication. The lack of betweenness centrality between the healthcare providers reflects this.

The main conclusion based on Figure 12 and Figure 14, and Table 12 is that information processing in every step goes only in one direction. There is a lack of closed loops and control mechanisms. This means that not only have the individual healthcare workers caused an enactment from a one-organ disease to a system disease, but they also lacked a feedback mechanism to warn them for the deterioration of the clinical condition of the patient. In order to reduce the discrepancy between the entropy of the body, requisite variety, robustness and resilience of the healthcare system and to prevent enactments like these, we should increase the robustness and resilience of the healthcare system, for example, by improving the information processes within the system, as explained by Galbraith [10].

**Figure 15:**
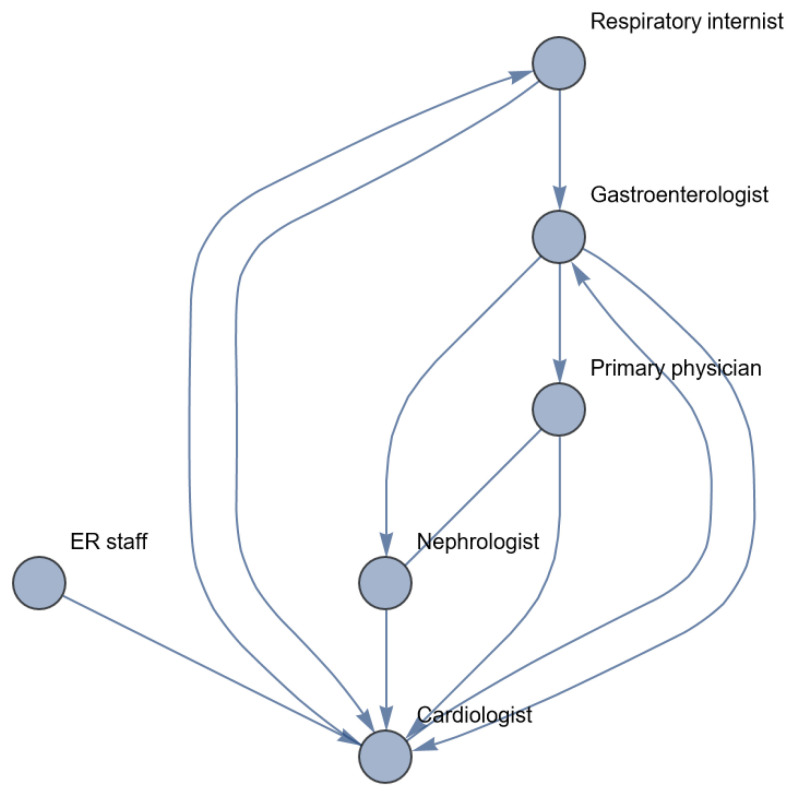
Healthcare network in Karl Weick’s case after investment in vertical relations.

A well-designed information-processing system may provide a timely diagnosis of the patient’s declined health, for example, a network in which the cardiologist is involved during every step of treatment and every change in the patient’s health state (see Figure 15 and its metrics in Table 13).

## 5. Discussion and Conclusions

In this study, we represent the human body and the healthcare system as complex networks and use concepts from theories regarding entropy, requisite variety and centrality metrics with resilience to quantify the strengths and weaknesses of healthcare systems. This provides tools to measure and improve the position and characteristics of the healthcare providers in a network, leading to important features involved in the quality and safety of healthcare. If the entropy of the patient’s health increases, the requisite variety of the healthcare system should increase equally (or more) in order to keep up with the patient conditions and to accurately diagnose and treat the patient. A healthcare system with high robustness and resilience is a system that accurately recognizes, adapts and intervenes with changes in a patient’s health state despite possible disruptions of the healthcare system, and is able to prevent or recover mistakes in the diagnostic and treatment phase. The theories of Weick and Galbraith can be applied to increase the robustness and resilience and thereby the quality and safety of a healthcare system.

### 5.1. Interpretation of Results and the Level of Analysis

To apply this theory to medical cases, we have to translate qualitatively described cases into quantitative tables. We were forced to categorize and simplify reality and to make assumptions. Depending on the case, the level of analysis can be adjusted (e.g., from microstate to macrostate). For example, if the goal is to visualize and improve the collaboration between medical specialists from different specialties, one could choose to analyze the case on an organ system level. If the goal is to visualize and improve the collaboration between co-workers from one discipline, one could analyze the case on a more detailed level, for example, per organ (instead of an entire organ system), per organ function, or even on a cell level.

In the theoretical example (Section 3, Figure 1) we visualized a network with nine elements, of which five were diseased (red): {1,2,3,4,5,6,7,8,9}. Suppose this network consists of multiple systems (the macroscopic level). One element could belong to multiple systems. Let us assume that this network consists of four systems: Red {1,2}, Green {3,4,5}, Blue {5,6,7} and Orange {7,8,9}. Indicated in the network: {{1,2},{3,4,{5},6,{7},8,9}}. The microscopic level is {1,2,3,4,5,6,7,8,9}. The macroscopic level is {red, green, blue, orange}. Microscopic changes might occur without observable changes on system level. A microscopic change within a system state may in- or decrease the chances of changing into another system state since an unaffected element of an affected system might potentially fulfill an important bridging function. The calculated betweenness centrality reflects the impact of the chosen level of detail on the outcome of the analysis.

In the case used by Karl Weick, we analyzed the case on a more macroscopic level. By translating the case into a quantitative table, we did not differentiate between affected organ systems and the underlying elements (organs and their separate functions). This represents the organ systems as a symmetrical, directed network (in which intermediate nodes are not necessary). If we were interested in analyzing a more detailed level, we should have defined the system states on a more microscopic level, visualizing how each organ system includes multiple organs (functions) and each organ may be part of multiple organ systems. The illness of one organ may induce sickness in other organs (systems) via central, bridging elements, which may activate a cascade of affected organs.

After the choice of the level of analysis, the level of analytical detail depends on the available information (based on the medical file) and the individual variety converting the medical file into the table. Individual interpretations may differ if detailed information about, for example, thoughts, differential diagnoses or consultations is missing in the patient database. This is an important limiting factor for analyzing cases in retrospect. Another limitation is the impossible measurement of the agents’ availability or reliability based on this description. To obtain uniformity and avoid arbitrariness, we recommend performing the conversion via at least two (medical) specialists.

### 5.2. Applicability of This Method

In the future, applying our theories may be used for the (legal) settlement after a medical error occurred. Currently, after a medical incident, a root cause analysis is usually performed, often with the aim of explaining what happened in terms of linear cause–effect relationships [20], leading to retrospectively judging the acts of individual healthcare providers. This approach is not in line with medical practice, in which teamwork and cooperation is inescapable. Another popular approach to analyze medical incidents as a whole is the Functional Resonance Analysis Method (FRAM) [27]. This method provides a qualitative system analysis on a very detailed, microstate level. Our method, on the other hand, provides a quantitative analysis of process organization and design level to determine the complexity of a case without having to know all the details of the patient’s health state and the performance of the individual agents involved. Usually, as a response to incidents, healthcare organizations create extra safety measures, like extra protocols, guidelines and care pathways. For standard situations, protocols are helpful, but the diversity and variety of patients, symptoms and diseases asks for professional decisions based on more than standard guidelines and protocols. In fact, the overload of protocols and guidelines may hinder or paralyze the professional detailed observations, creative thinking and clinical performances. It may undesirably lead to ‘defensive medicine’, with redundant (over-) diagnostics and (over-) treatments. Even a whole system of ‘super doctors’ and other health personnel would not lead to a healthcare system of high quality if the cooperation and information processing between the super doctors are not properly organized.

Building a strong professional network is very important for the quality and safety of the medical care and may prevent the occurrence of adverse events. Applying our theories and methods to medical cases in which errors or adverse events occurred may lead to valuable insights into the strengths and weaknesses of a healthcare system and the agents within the system. These quantified metrics have to be the starting point for a reorganization of the healthcare system in order to prevent future errors. Possible changes to the healthcare system (such as the investment in information technology, vertical or lateral relations) can be quantified using resilience and entropy metrics. This helps to predict the effect of possible changes in the healthcare organization and should be considered when care pathways are established.

Finally, analyzing the healthcare system this way may provide other insights for healthcare organizations, for example, information to manage challenges like reduced availability of healthcare providers or resources. During the COVID-19 pandemic, alternative patient routings were developed. Our method could be used to measure the impact of organizational design changes on healthcare safety and efficiency to be sure that changes do not (further) increase the healthcare system’s burden.

### 5.3. Practical Aspects and Limitations

#### 5.3.1. The Uncertainty Regarding the Uncertainty—Entropy

There are limitations on this method as well. With the benefit of hindsight, it might be obvious when a change in health state or an error occurred. When translating a qualitatively described case into quantitative tables, it is important to follow the healthcare provider(s) steps and trains of thought, differential diagnoses, and diagnostic and treatment steps in order to discover weaknesses in the healthcare as provided. After all, entropy is always in the eye of the beholder. When calculating the entropy regarding the true microstate causing a macrostate, entropy is highest (maximal entropy) when all possible underlying microstates (this is the differential diagnosis the doctor makes when they are confronted with the patient) have the same probability of declaring the macrostate [31].

In medical practice, the doctor will prioritize the differential diagnosis based on the likelihood of a priori changes, family history, medical history, comorbidities, etc. This means that the possible microstates will be weighted based on occurrence, and entropy will be less than that calculated with non-weighted chances of occurrence. In network terms, this means that the probability distribution is not uniform but exponentially distributed. This is the result of patient-specific constraints [40]. It is important to have constraints: constraint lends itself as a most useful measure of the degree of order, rigidity, or regulation that exists in an organization. Medical literature, protocols and expert opinions are necessary to calculate a weighted entropy.

#### 5.3.2. Concurrence with Other (Patient) Paths

Another limitation of this method is the focus on one patient(path). In practice, most agents and resources will be involved in the treatment of several patients at the same time. Professional decision making and the availability of resources could be influenced because of that, as Van Merode et al. have shown: different patient care trajectories are connected through shared resources, and the coordination of this is necessary to avoid systemic failures appearing when local blockages result in further sequences of local blockages [41]. This means that even if all the healthcare professionals are fully competent and informed from a medical expertise point of view, the healthcare system might still fail. To overcome this limitation, an additional analysis at a more macroscopic level, including multiple patient paths, could be performed.

### 5.4. Future Use and Research

Our method can be immediately used to develop safe and efficient care pathways. This analysis provides information about the robustness and resilience of the healthcare system and the impact of horizontal and lateral relationships (information processes). Medical incidents should be analyzed in this way because it considers complex systems and provides more information than the usual methods, visualizing the impact of the system and cooperation within a network on the occurrence of adverse events or errors. In addition, this method may form a valuable addition to current methods of legal evaluation and judgement after adverse events or incidents.

A possible next step could be the use of this method to calculate business aspects, such as the financial consequences of diverse organizational designs.

## Figures and Tables

**Table 1:** The relation between disease stage and required requisite variety to recover.

Disease Stage ID	Disease Stage	Disease Stage Characteristics
0	Healthy	No symptoms
1	Non-healthy	Requisite variety of own body activated
2	Non-healthy single organ	Requisite variety of one specific medical specialist needed
3	Non-healthy multiple organ	Requisite variety of a team of medical specialists needed

**Table 2:** Entropy of the human health during different health states, developing from a healthy stage to a multiple organ disease and to a systemic disease.

System State	Entropy
Healthy	3.74
State 1	3.28
State 2a	3.04
State 2b	2.58
State 3	1.00
Final	0.00

**Table 3:** Centrality metrics of the agents in the previous example and network entropy.

Metrics					
Agent	Doctor 1	Doctor 2a	Doctor 2b	Doctor 2c	Doctor 3
Vertex in-degree	0	3	3	3	4
Closeness centrality	1	1	1	1	0
Betweenness centrality	0	0	0	0	0
Entropy	2.59				

**Table 4:** Centrality metrics of the agents in the previous example and network entropy after investment in vertical information systems.

Metrics after Investment in Vertical Relations					
Agent	Doctor 1	Doctor 2a	Doctor 2b	Doctor 2c	Doctor 3
Vertex in-degree	4	2	1	1	1
Closeness centrality	1.0	0.57	0.57	0.67	0.57
Betweenness centrality	11	0	0	0	0
Entropy	2.17				

**Table 5:** Centrality metrics of the agents in the previous example and network entropy after investment in lateral information systems.

Metrics after Investment in Lateral Relations			
Agent	Doctor 1	Pooled Subspecialists (2a + 2b + 2c)	Doctor 3
Vertex in-degree	0	1	2
Closeness centrality	1	1	0
Betweenness centrality	0	0	0
Entropy	1.17		

**Table 6:** Centrality metrics of the agents in the previous example and network entropy after investment in lateral and vertical information systems.

Metrics after Investment in Lateral and Vertical Relations			
Agent	Doctor 1	Group Subspecialists (2a + 2b + 2c)	Doctor 3
Vertex in-degree	1	1	1
Closeness centrality	1	0.67	0
Betweenness centrality	1	0	0
Entropy	0.92		

**Table 7:** An experimental design table, showing the impact of the availability (*A_i_*) and reliability (*R_i_*) (scored with values of 1 (low), 3 (average) or 5 (high)) of each agent from the last example (the combination of lateral and vertical relations) on their resilience (as shown in Table 6).

Node	Availability	Reliability	Total Resilience Score
Doctor 1	1	1	1
	1	3	3
	1	5	5
	3	1	3
	3	3	9
	3	5	15
	5	1	5
	5	3	15
	5	5	25
Group subspecialist	1	1	0.67
	1	3	2
	1	5	3.33
	3	1	2
	3	3	6
	3	5	10
	5	1	3.33
	5	3	10
	5	5	16.67
Doctor 3	1	1	0
	1	3	0
	1	5	0
	3	1	0
	3	3	0
	3	5	0
	5	1	0
	5	3	0
	5	5	0

**Table 8:** Legends of organ systems involved in this case.

Organ System	Abbreviation
Integumentary system	O1
Skeletal system	O2
Muscular system	O3
Nervous system	O4
Endocrine system	O5
Cardiovascular system	O6
Lymphatic system	O7
Respiratory system	O8
Digestive system	O9
Urinary system	O10
Reproductive system (male/female)	O11

**Table 9:** Legends of system states involved in this case.

System State	Abbreviation
A patient with one organ system affected (O6)	S1
A patient with two organ systems affected (O6 and O8)	S2
A patient with three organ systems affected (O6 and O8 and O9)	S3
A patient with four organ systems affected (O6 and O8 and O9 and O10)	S4
A patient with a systemic disease	S5
A dead patient	S6

**Table 10:** Translation of the medical case into a table, representing the situations described by health state, agents, resources and differential diagnosis.

Time	State	Agent	Resource	Connection	Applied to Agent	Outcome	Diff. Diagnosis
Day 1	S1	A1	R0	C1	A1	S1	D1
Day 3	S1	A1	R1	C2	A2	S1	D1
Day 3	S1	A2	R1	C1	A1	S1	D1
Day 3	S1	A2	R2	C3	A1	S1	D1
Day 3	S1	A3	R0	C1	A1	S1	D1
Day 3	S1	A3	R2	C3	A1	S1	D1
Day 3	S1	A3	R101	C4	A1	S1	D1
Day 3	S1	A1	R101	C4	A3	S1	D1
Day 3	S1	A1	R101	C5	A3	S1	D1
Day 3	S1	A4	R0	C6	A1	S1	D1
Day 3	S1	A10	R13	C7	A4	S1	D1
Day 3	S1	A4	R13	C7	A1	S1	D1
Day 3	S1	A4	R5	C8	A1	S1	D1
Day 3	S1	A4	R0	C9	A1	S1	D1
Day 3	S1	A4	R101	C4	A1	S1	D1
Day 3	S1	A1	R101	C4	A4	S1	D1
Day 3	S1	A1	R101	C5	A4	S1	D1
Day 3	S1	A4	R3	C10	A1	S1	D1
Day 3	S1	A4	R4	C11	A1	S1	D1
Day 3	S1	A5	R101	C4	A1	S1	D2
Day 3	S1	A1	R101	C4	A5	S1	D2
Day 3	S1	A1	R101	C5	A5	S1	D2
Day 3	S1	A5	R6	C10	A1	S1	D2
Day 3	S1	A5	R7	C10	A1	S1	D2
Day 3	S1	A5	R8	C10	A1	S1	D2.D3
Day 3	S1	A5	R1	C12	A6	S2	D2.D3
Day 4	S2	A11	R100	C16	A1	S2	D2.D3.D4.D5.D6
Day 3	S1	A6	R9	C10	A1	S1	D2.D3.D4
Day 4	S2	A11	R100	C16	A1	S2	D2.D3.D4.D5.D6
Day 4	S2	A5	R6	C13	A1	S2	D2.D3.D4
Day 4	S2	A5	R1	C12	A7	S3	D2.D3.D4
Day 4	S3	A11	R100	C16	A1	S3	D2.D3.D4.D5.D6
Day 4	S3	A7	R10	C14	A1	S3	D2.D3.D4.D5
Day 4	S3	A7	R11	C10	A1	S3	D2.D3.D4.D5
Day 4	S3	A11	R100	C16	A1	S3	D2.D3.D4.D5.D6
Day 4	S3	A1	R3	C13	A1	S3	D2.D3.D4.D5
Day 4	S3	A1	R6	C13	A1	S3	D2.D3.D4.D5
Day 4	S3	A1	R7	C13	A1	S3	D2.D3.D4.D5
Day 4	S3	A1	R8	C13	A1	S3	D2.D3.D4.D5
Day 4	S3	A1	R9	C13	A1	S3	D2.D3.D4.D5
Day 4	S3	A1	R10	C13	A1	S3	D2.D3.D4.D5
Day 4	S3	A11	R100	C16	A1	S3	D2.D3.D4.D5.D6
Day 4	S3	A8	R1	C2	A9	S4	D2.D3.D4.D5
Day 4	S4	A9	R12	C15	A1	S4	D2.D3.D4.D5
Day 4	S4	A9	R13	C9	A1	S5	D2.D3.D4.D5.D6
Day 4	S5	A11	R100	C16	A1	S5	D2.D3.D4.D5.D6
Day 4	S5	A11	R100	C16	A1	S5	D2.D3.D4.D5.D6
Day 4	S5	A1	R100	C0	A1	S6	D2.D3.D4.D5.D6

**Table 11:** Entropy of the human body in each system state.

System State	Entropy
A healthy patient (0 organ systems affected)	4.92
A patient with one organ system affected	4.64
A patient with two organ systems affected	4.34
A patient with three organ systems affected	4.00
A patient with four organ systems affected	3.61
A patient with a systemic disease	3.17
A dead patient	0

**Table 12:** Centrality metrics of the agents in Karl Weick’s case and network entropy.

Healthcare System						
	ER Staff	Cardiologist	Respiratory Internist	Gastroenterologist	Primary Physician	Nephrologist
Vertex in-degree	0	1	1	2	1	1
Closeness centrality	0.45	0.67	0.60	1.0	1.0	1.0
Betweenness centrality	0	0	0	0	0	0
Entropy	2.94					

**Table 13:** Centrality metrics of the agents in Karl Weick’s case and network entropy after investment in vertical relations.

Healthcare System with Vertical Relations						
	ER Staff	Cardiologist	Respiratory Internist	Gastroenterologist	Primary Physician	Nephrologist
Vertex-indegree	0	5	1	2	1	1
Closeness centrality	0.45	0.67	0.67	0.8	0.67	0.67
Betweenness centrality	0	4.5	0	0	0.5	0.5
Entropy	2.78					

## Data Availability

No new data were created or analyzed in this study. Data sharing is not applicable to this article.

## Appendix A. Key Concepts and Definitions

### Appendix A.1. Order and Disorder

An order is the arrangement or disposition of people or things in relation to each other according to a particular sequence, pattern or method. By ‘order’, the organization, structure and function of an arrangement are meant. What is classified as ‘order’ depends on definitions and is related to the classification of certain conditions. A disorder is a state of confusion compared to the state of order. Order and disorder are defined by the numbers of ways in which a system can be configured [42].

### Appendix A.2. Graph Theory

Network theory is a subset of graph theory, a branch of mathematics concerning the visual representation of set of objects and the links connecting pairs of objects [43].

A network is a catalog of the components of a system, often called nodes or vertices ( k ) and edges (*L*). Nodes/vertices represent objects (for example, the people who deliver health, their resources or supplies). Edges are the lines that connect the nodes, representing the association between them [21]. These associations (links) can be directed or undirected, and weighted or unweighted [44].

Network theories enable the computation of centrality metrics that reveal the importance of a node in a network. For example, a node high on *degree centrality* is one with many edges connecting it to other nodes. A node high on *strength centrality* in a network is one that has many edges connected to it that are great in terms of their magnitude of associations [21]. The *degree* $k_{i}$ of a node (an agent or a resource) is the number of links it has to other nodes [12]. If we consider all links undirected, the total number of links (L ) is the sum of the node degrees:

$$L=\frac{1}{2}\overset{N}{\underset{i=1}{\sum }}k_{i}$$

The most natural complexity measures are the order of the graph, meaning the number (*n*) of the nodes, and the size of the graph, meaning the number of the edges (*m*). The number of edges incident to vertex (*v)* is the degree of the vertex, *deg(v)*.

### Appendix A.3. Fitness of Nodes

In graph theory, each node has a certain degree of fitness, reflecting its position in the network and the connectivity of the node. Different centrality parameters can be used to measure the degree of fitness of a node [28,45].

In social network analysis, the following centrality metrics are often used at an individual level of the actors: in-degree, closeness and betweenness. The in-degree centrality corresponds to the count of the number of ties directed to the node. The betweenness centrality corresponds to the number of node pairs that a given node connects. Actors with high betweenness scores are positioned at the crossing of multiple shortest paths connecting individuals in a network. The closeness centrality indicates how close a node is to all others in the network; it is given by the average of the shortest path length from that node to every other node in the network. A low value indicates that a player is more reachable by others [30].

### Appendix A.4. Entropy

Entropy is a basic understanding, founded in thermodynamics. *Boltzmann* formulated the statistical definition of entropy for systems that can exchange thermal energy with the surroundings. Molecular systems are composed of many interacting atoms. If the number of atoms in a macromolecular system increase, the thermodynamic observables increase as well. The macroscopic state of a system is called a *macrostate*. A *microstate* (also called ‘multiplicity’) is a specific configuration of all the constituent atoms that make up the system. Multiple microstates can configure the same macrostate.

Boltzmann defined the entropy (S ) as a function of the number of microstates consistent with the given macrostate (also named multiplicities) ( W) as follows:

$$S=K_{b}\mathrm{In}\;W$$

in which K_b_ is Boltzmann’s constant equal to 1.38062 × 10^−23^ joule/Kelvin.

Boltzmann’s definition allows us to consider entropy from the perspective of the probabilities of different configurations (microstates) of the constituent interacting particles in an ensemble (macrostate). More easily formulated, it provides a measure of how many microstates would produce the same microstate, and therefore of how likely the state is. The definition was formulated for systems that can exchange thermal energy with the surroundings but may be applicable to other sciences as well, for example, information theory. *Shannon* (1948) defined entropy for information theory as the amount of ‘uncertainty’ present in a variable and aimed to mathematically measure the statistical nature of lost information in communication. His theory defines a data communication system composed of three elements: (1) a source of data, (2) a communication channel, and (3) a receiver. The fundamental problem of communication is for the receiver to be able to identify what data were generated by the source based on the signal it receives through the channel. Shannon has expressed the relationship between the probability and the heterogeneity in the mathematical form using the following equation:

$$Entropy\;\left(p\right)=-\overset{N}{\underset{i=1}{\sum }}p_{i}\log_{2}p_{i}$$

In this equation, the uncertainty is represented as the log to base 2 of the probability of a category (*p_i_*). The index (*i*) refers to the number of possible categories. In case a ‘problem’ is a binary classification, the index is 2. In other situations, the index may increase.

The entropy of the communication system from Shannon represents an absolute mathematical limit on how well data from the source can be compressed onto a perfectly noiseless channel. Events with higher uncertainty have higher entropy.

### Appendix A.5. Requisite Variety

Once a system becomes ‘out of control’ (changes from order to disorder), requisite variety is what is needed to bring the system back to normal (back to the state it was before the changes) [7]. So, given a disturbance or input, a system is assumed to respond to the disturbance with a regulatory process or action, which, in turn, leads to an outcome (as preferred, back to normal, back to ‘order’). The law of requisite variety states that the system that regulates the possible responses to disturbances must match the number of states in the input, with at least an equivalent number of states in the response process [9].

### Appendix A.6. Negentropy

Negentropy is reverse entropy and means becoming more ‘in order’. In communication, it may also be described as the statistical measure of the probability of meaningful information and the probability of ‘order’. Schrödinger [46] uses the term negentropy to identify the remarkable ability of a living system to increase organization in order to deal with entropy and uncertainty (or disorder). Negentropy describes how some systems are capable of generating order, complexity, and life from chaos, disorder and entropy. Thus, negentropy refers to the ability of some systems to store information and energy and use them to *maintain* their organization by performing useful work.

### Appendix A.7. Resilience and Negentropy

Resilience is a quality of a system and is based on the ability of a system to cope with disruptive events. If a system becomes disturbed (with perturbations from in- or outside the system), the system’s state can change from *order* to *disorder*. The resilience of a system denotes how quickly a system recovers after perturbations and what is needed to get back to ‘order’. Highly connected and homogeneous networks tend to be more resilient to change. The ability of a network to compensate from perturbations depends on the structure of the network. There is no unanimous or universal indicator or way to measure the resilience of a system. Duran et al. [17] explored the field of (neg)entropy in the search for a general resilience indicator/measuring tool.

Negentropy is, according to Duran, a well-suited indicator for evaluating resilience in engineering systems due to its advantageous characteristics: (1) it has the ability to maintain functionality and adaptability in the face of disruptive events; (2) it offers a holistic perspective by considering the system as a whole, taking into account the interdependencies and interactions between its components; and (3) it is sensitive to changes in the system’s behavior and performance over time, enabling it to capture variations and fluctuations in its response to disruptions, thus reflecting its adaptive capacity. Negentropy can by calculated using an equation, derived from Shannon’s entropy equation and facilitates the development of quantitative metrics and indices.

The higher the negentropy of a system measured from the analysis of the behavior over time of some performance variable, the higher its resilience.

The negentropy (*N*) *=* (1/*H*), in which *H* is Shannon’s entropy.

### Appendix A.8. Enactment

Karl Weick (1988) uses the term *enactment* as representing the notion that when people act, they bring structures and events into existence and set them in motion. When people act, they unrandomize variables, insert vestiges or orderliness, and create their own constraints [38].

## Appendix B. Conversion Tables

**Table A1:** Conversion table agents.

Agent	Abbreviation
Patient	A1
Telephonist emergency center	A2
Ambulance staff	A3
ER staff	A4
Cardiologist	A5
Respiratory internist	A6
Gastroenterologist	A7
Primary physician	A8
Nephrologist	A9
Lab technicians	A10
Nurses IC	A11

**Table A2:** Conversion table resources.

Resources	Abbreviation
Self-capability	R0
Telephone	R1
Ambulance	R2
Infusion Pump	R3
Intubation supplies	R4
ECG supplies	R5
Heparin (drug)	R6
Lidocaine (drug)	R7
Pronestyl (drug)	R8
Theophylloine (drug)	R9
Gastroenteroscopy supplies	R10
Pepcid (drug)	R11
Medical file	R12
Laboratory supplies	R13
ICU bed	R100
Communication device	R101

**Table A3:** Conversion table connections.

Connections	Abbreviation
Makes risk assessment	C1
Calls	C2
Sends	C3
Communicates	C4
Provides informed consent	C5
Physical examination	C6
Runs blood tests	C7
Runs ECG	C8
Diagnosis	C9
Infusion of medication	C10
Intubates	C11
Consults	C12
Causes side effects	C13
Gastroenteroscopy	C14
Checks medical file	C15
Nurses	C16

**Table A4:** Conversion table differential diagnoses.

Diff. Diagnosis	Abbreviation
Heart attack	D1
Myocardial infarction	D2
Arrhythmia	D3
Pulmonary edema and risk of bronchospasm	D4
Stress gastritis	D5
Dilution hyponatremia	D6
