# Correction: Klemann et al. Quantifying the Resilience of a Healthcare System: Entropy and Network Science Perspectives. *Entropy* 2024, *26*, 21

**DOI:** 10.3390/e27050519

**Published:** 2025-05-13

**Authors:** Désirée Klemann, Windi Winasti, Fleur Tournois, Helen Mertens, Frits van Merode

**Affiliations:** 1 Department of Gynecology and Obstetrics, Maastricht University Medical Centre+, Maastricht University, 6229 HX Maastricht, The Netherlands; fleur.tournois@mumc.nl; 2 Care and Public Health Research Institute, Maastricht University, 6200 MD Maastricht, The Netherlands; f.vanmerode@maastrichtuniversity.nl; 3 IQ Healthcare, Radboudumc, 6525 EP Nijmegen, The Netherlands; w.winasti@etz.nl; 4 Elisabeth-TweeSteden Ziekenhuis, 5022 GC Tilburg, The Netherlands; 5 Executive Board, Maastricht University Medical Centre+, 6229 HX Maastricht, The Netherlands; helen.mertens@mumc.nl; 6 Maastricht University Medical Centre+, 6229 HX Maastricht, The Netherlands

In the original publication [1], there were mistakes in Figure 11 and Table 11 as published. The system state for a healthy patient was mistakenly omitted; as a result, the entropy values for some system states were incorrect in the table and figure. The corrected Figure 11 and Table 11 appear below.

The authors state that the scientific conclusions are unaffected. This correction was approved by the Academic Editor. The original publication has also been updated.

## Figures and Tables

**Figure 11:**
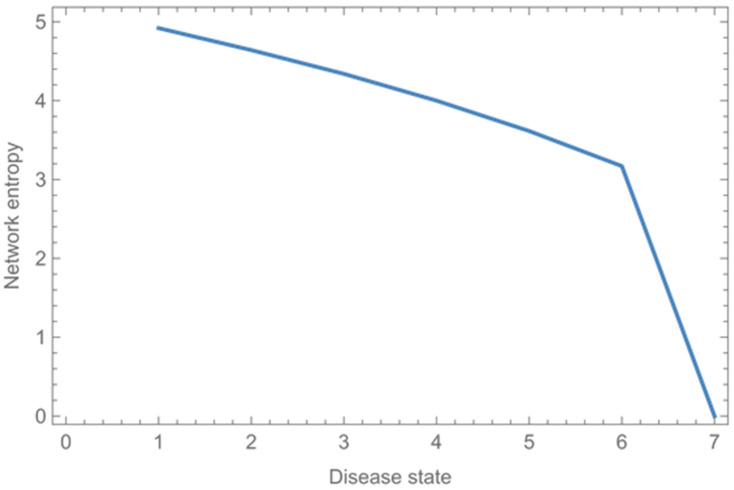
Entropy of the human body in each system state.

**Table 11:** Entropy of the human body in each system state.

System State	Entropy
A healthy patient (0 organ systems affected)	4.92
A patient with one organ system affected	4.64
A patient with two organ systems affected	4.34
A patient with three organ systems affected	4.00
A patient with four organ systems affected	3.61
A patient with a systemic disease	3.17
A dead patient	0
